# Non-Communicable Disease Risk Factors among Employees and Their Families of a Saudi University: An Epidemiological Study

**DOI:** 10.1371/journal.pone.0165036

**Published:** 2016-11-04

**Authors:** Rasmieh Alzeidan, Fatemeh Rabiee, Ahmed Mandil, Ahmad Hersi, Amel Fayed

**Affiliations:** 1 King Saud University, College of Medicine, Cardiac Sciences Department, Riyadh, Saudi Arabia; 2 Birmingham City University, School of Health Sciences, Faculty of Health, Education and Life Sciences, Birmingham, United Kingdom; 3 King Saud University, College of Medicine, Family and Community Medicine Department, Riyadh, Saudi Arabia; 4 Princess Nourah Bint Abdulrahman University, College of Medicine, Riyadh, Saudi Arabia; 5 Alexandria University, High Institute of Public Health, Biostatistics Department, Alexandria, Egypt; University of Oxford, UNITED KINGDOM

## Abstract

**Objectives:**

To assess the prevalence of noncommunicable disease (NCD) risk factors among Saudi university employees and their families; to estimate the cardiovascular risk (CVR) amongst the study population in the following 10years.

**Methods:**

The NCD risk factors prevalence was estimated using a cross-sectional approach for a sample of employees and their families aged ≥ 18 years old, in a Saudi university (Riyadh in Kingdom of Saudi Arabia; KSA). WHO STEPwise standardized tools were used to estimate NCD risk factors and the Framingham Coronary Heart Risk Score calculator was used to calculate the CVR.

**Results:**

Five thousand and two hundred subjects were invited, of whom 4,500 participated in the study, providing a response rate of 87%. The mean age of participants was 39.3±13.4 years. The majority of participants reported low fruit/vegetables consumption (88%), and physically inactive (77%). More than two thirds of the cohort was found to be either overweight or obese (72%), where 36% were obese, and 59% had abdominal obesity. Of the total cohort, 22–37% were found to suffer from dyslipidaemia, 22% either diabetes or hypertension, with rather low reported current tobacco use (12%). One quarter of participants was estimated to have >10% risk to develop cardiovascular disease within the following 10-years.

**Conclusion:**

The prevalence of NCD risk factors was found to be substantially high among the university employees and their families in this study.

## Introduction

Non-communicable diseases (NCDs), mainly cardiovascular diseases (CVDs), were reported to be responsible for 64% of the global deaths during 2014 [1], more than 80% of deaths occurred in developing countries [1]. In Eastern Mediterranean region every year 1.7 million people die from the four main NCDs (CVDs, cancer, chronic respiratory and diabetes), if no serious action is taken, the deaths due to NCDs is projected to reach 2.4 million in 2025 [2]. Moreover, Eastern Mediterranean region is recognized as the hot-spot of NCD particularly CVDs and diabetes [3]. The trends of NCD showed variations between the countries of Eastern Mediterranean region of different income levels, and where the low income countries are still grappling with communicable diseases, while the tide of NCDs is increasing. Given that the rate of deaths from NCDs in 2014 was ranging from 19% in Somalia to 85% in Egypt and Lebanon [1]. Likewise, Gulf Cooperation Council (GCC) countries have witnessed a higher burden of NCD mortality which ranged from 65% to 78% of the total adult deaths [1].

NCDs are considered the primary cause of death among adults, being implicated for most (78%) of the total deaths in Kingdom of Saudi Arabia (KSA) during 2014, of which CVDs were responsible for about (46%) of these deaths [4]. KSA was exposed to a vast epidemiological transition, rapid economic development and ageing population. Subsequently, major modifications have happened in lifestyle behaviours such as: increased caloric and high fat-diet consumption; reduced physical activity and dramatic increase in obesity prevalence among different age-groups. This has all lead to increased prevalence of NCDs, including diabetes mellitus and CVDs [5].

Employees in any institution represent an important population category; their quality of life, health awareness and ability to embrace healthy behaviors are expected to influence their productivity, avoid NCD occurrence, reduce healthcare costs and as a result improve the economic status of the workplace [6].To our knowledge and to-date, there is no large-scale study examining NCD risk factors in employees and their families in KSA[7–11], hence this study focused on this population group.

Conventional risk factors of NCD are classified into two groups: behavioural NCD risk factors as tobacco use, physical activity and dietary pattern, and clinical NCD risk factors as obesity, hypertension, diabetes mellitus and hyperlipidemia [12]. These risk factors have exhibited clustering, and synergizing over time, and association with higher prevalence of NCD, including cardiovascular events and related mortality [13].

The objectives of this study were to examine the NCD risk factors prevalence among King Saud University employees and their families, to assess the rate of clustering of risk factors amongst the study population and to estimate the cardiovascular risk in the following ten years

## Materials and Methods

### Study setting, design and population

King Saud University is the oldest university in Kingdom of Saudi Arabia, which includes 40 colleges (health, non-health). Moreover, under the umbrella of King Saud University, there are two teaching hospitals, namely: King Abdul-Aziz University Hospital (ophthalmology and ENT) and King Khalid University Hospital, which is a tertiary referral hospital with a 1000-bed capacity. In 2013, King Saud University’s employees were 25,125, of whom 77% are Saudis and 23% expatriates [14].

A cross- sectional approach was used for carrying out this study at King Khalid University Hospital, for a period of 9 months (from 8 July, 2013 to 30 April, 2014). The primary healthcare clinics for employees and their families in King Khalid University Hospital include: two clinics for university staff, and a third to serve hospital staff. The WHO STEPwise approach to surveillance of NCD risk factors was used in our study [15]. We invited 5,200 subjects to our study were 4500 participants agreed to be enrolled in this study (response rate of 87%). A convenience sample of 4,500 employees and their families aged ≥18 years (family in this context referred to: spouses, children and employee’s parents) were enrolled for this study’s purposes; pregnant women and subjects aged less than 18 years were excluded.

We based our sample size calculation to detect the lowest prevalence (1.5%) of CVDs, as has been published in Riyadh city (2011) [16], with 0.5% precision and 95% confidence. The minimum of 4250 participants was needed to reject the null hypothesis at power of 80%. To assure proper distribution of the collected sample, we recruited 2250 employees and 2250 of their families.

### Data collection

A modified form of WHO STEPS questionnaire, version 2.1 (both Arabic and English forms) was used in this study, after pilot testing on 30 subjects (who were excluded from analysis). This questionnaire uses sequential steps, starting with a questionnaire (Step I), physical measurements (Step II) followed by biochemical measurements (Step III) [17]:

#### Step I

Self-reported questionnaire: All participants were asked to self-complete the STEPS survey study tool which enquired about: socio-demographic information (including: gender, age, level of education, marital status, nationality and job title); behavioral habits (including: tobacco use, fruit/vegetable consumption, physical activity), as well as their medical history which specifically enquired about history of raised blood pressure and/or diabetes.

#### Step II

Anthropometric measurements: weight and height were measured for all participants barefooted, with relaxed shoulders and arms hanging freely, using a digital weighing scale (SECA 220; Hamburg, Germany 2009), which was re-calibrated before each use. Weight was measured to the nearest 10 grams, while height was measured to the nearest 0.1 cm. The Body Mass Index (BMI) was calculated using the formula BMI = weight (kg) /height (m^2^). Based on the BMI, the study population was divided into five groups: underweight, normal weight, overweight, obese and morbidly obese: <18.5; 18.5–24.9; 25–29.9; 30–34.9 and ≥35, respectively, as these cut-off values for BMI classification were reported to be applicable to Arab nations. Nevertheless, these values were adjusted for the South Asian population of this study, with the cut-offs for BMI being: <18.5, 18.5–23, 23.1–27.5, 27.6–32.5, and >32.5, respectively [18].

The waist circumference (WC) was measured in centimeters to the nearest 0.1 cm, using a flexible non-stretchable plastic tape, in a standing relaxed position, during expiration, at the midline between the lower costal margins and the iliac crest parallel to the floor. A WC of ≥88 cm and ≥102 cm were used for diagnosis of abdominal obesity (AO) among women and men, respectively, which are cut-off values reported to be applicable to Arab ethnicities [19]. The South Asian subjects were identified as having AO if WC > 80 for women and >90 for men [20]. The waist height ratio (WHtR) was calculated by dividing WC (in cm) by height (in cm). WHtR ≥ 0.5 indicated AO for women and men for all ethnic groups [21].

After 10 minutes rest, arterial blood pressure was measured using an automated standard digital sphygmomanometer (Mindary Datascope Accutorr Plus, USA 2010), placed on the subject's left arm during a relaxed and sitting position. Both systolic and diastolic pressures were measured, at two readings, set five minutes apart; the average of the two readings was used.

#### Step III

Biochemical tests: The biochemical measurements in this study included: glycosylated heamoglobin (HBA1C), high density lipoprotein-cholesterol (HDL-C), low density lipoprotein-cholesterol (LDL-C), total cholesterol (TC) and triglycerides. Participants were instructed to fast for at least 12 hours before being subjected to blood testing.

#### Cardiovascular risk scores

CVR scores were calculated for all participants on weekly basis using Framingham Coronary Heart Risk Score, then cohorts were sub-divided according to their scores into three categories: low risk score (<10%), intermediate (10–20%), and high risk score (>20%) [22].

### Definitions

Diabetes mellitus was defined as per WHO and American Diabetes Association criteria, or by subject reporting of being previously diagnosed as diabetic and using anti-diabetes medication [23].

Hypertension was defined as being previously diagnosed as hypertensive and currently using any anti-hypertensive medications or having high blood pressure readings according to JNC7 [24].

Subjects were categorized as having any sort of *dyslipidaemia* according to the criteria of WHO and the National Cholesterol Education Program (NCEP) [25], or by subject reporting of being on current lipid- regulating medications.

Smoking status: participants were identified as “current smokers” when reporting smoking at least one cigarette daily during the last 6 months, 1 cigar or water-pipe per week during the last 6 months, or one waterpipe tobacco smoke /shisha session per month during the previous 3 months. Participants who reported quitting smoking for a year or more were identified as “former smokers”, while those who reported that they never smoked were identified as “non-smokers” [26].

Physically inactivity was considered if a subject was not meeting any of WHO criteria: 60 minutes/week of vigorous–intensity activity or 150 minutes/week of moderate intensity activity. According to the WHO, any subject who had less than five servings (400gm) of fruit and/or vegetables per day was considered as having low fruit and vegetable intake [27].

### Statistical analysis

Data was entered using the Statistical Package for Social Sciences (SPSS), namely PASW statistics data document 18. Continuous variables, interval and ratio variables were reported as means with standard deviations. Categorical variables were presented as frequencies with equivalent percentages, a Fisher's Exact or Pearson’s χ2 tests for comparing different proportions. Logistic regression analysis was conducted to adjust for the age effect as a possible confounder of NCD risk factors among different subgroups.

The logistic regression model was used to adjust the possible effect of different socio-demographic variables on clustering of NCD risk factors. The dependent variable was “having three or more NCD risk factors”, while independent factors included: age, gender, educational level, marital status and occupation. Adjusted Odds Ratio and 95% Confidence Interval (CI) were used for interpretation of the adjusted risk, and p value < 0.05 was considered statistically significant.

### Ethical statement

The approval letter (number 13–3721) of King Saud University -Institutional Review Board (IRB) was sought prior to study commencement. The informed written consent was obtained from each participant before questionnaire completion and blood sample withdrawn. The study was conducted as per the principles expressed by Declaration of Helsinki.

## Results

### Socio-demographic characteristics

The overall mean age (±standard deviation; SD) was 39.3 (±13.4) years, where the Saudi group of participants was significantly younger than other nationalities (p = 0.001, **Table 1**). Just above two thirds of participants were Saudis 68%, 24% were Arab non-Saudis (ANS = Algerians, Egyptians, Jordanians, Moroccans, Palestinians, Syrians, Sudanese, Tunisians, and Yemeni). South Asians constituted 7% of the studied population, including Bengalis, Indians, and Pakistanis, while 1% (n = 37) from different nationalities all over the world, were excluded from analysis as they did not represent a specific group of countries. **Table 1** shows the socio-demographic characteristics distributed by nationality.

**Table 1:** Socio-demographic characteristics of participants by nationality (n = 4,500).

Characteristic	Saudis 3063 (68%)	ANS 1091 (27%)	South Asian 309 (7%)	p value
Age in years (mean ± SD)	38.58±14.09	40.67±11.8	40.38±11.22	0.001
**Gender**				
Males	1156(37.7)	647(59.3)	180(58.3)	<0.001
Females	1907(62.3)	444(40.1)	129(42.7)	<0.001
**Age group (in years)**				
18–29	1046(34.1)	190(17.4)	29(9.4)	<0.001
30–39	663(33.2)	362(33.2)	133(43.0)	<0.001
40–49	545(17.8)	280(25.7)	71(23.0)	<0.001
50–59	542(17.7)	175(16.0)	55(17.8)	<0.001
≥60	267(8.7)	84(7.7)	21(6.8)	<0.001
**Education**				
Higher education (college & above)	1870(61.1)	943(86.4)	291(94.2)	<0.001
Essential education	1035(33.8)	144(13.2)	18(5.8)	<0.001
Illiterate	158(5.2)	4(0.4)	0(0.0)	<0.001
**Participant status in university**				
Employee	1368(44.7)	722(66.2)	232(75.1)	<0.001
Family member	1695(55.3)	369(33.8)	77(24.9)	<0.001
**Occupation**				
Teaching staff	558(18.2)	402(36.8)	145(46.9)	<0.001
Healthcare provider	105(3.4)	38(3.5)	57(18.4)	<0.001
Technician	143(4.7)	7(0.6)	9(2.9)	<0.001
Administrative	295(9.6)	17(1.6)	7(2.3)	<0.001
Other university employees	267(8.7)	258(23.6)	14(4.5)	<0.001
**Marital status**				
Single	757(24.7)	85(7.8)	17(5.5)	<0.001
Married	2187(71.4)	996(91.3)	292(94.5)	<0.001
Widowed and divorced	119(3.9)	10(0.9)	0(0.0)	<0.001

Essential education = indicated a completion of any school: elementary, preparatory and high school Data are presented as mean± standard deviation (SD) or as number of subjects (precentage).

### Prevalence of behavioral NCD risk factors

#### Tobacco use

Overall, a rather low prevalence of current daily tobacco smoking was reported among the studied population (12%), with predominately manufactured cigarettes used more than waterpipe tobacco smokes (70% vs. 30% respectively). More details are shown in **Table 2**.

**Table 2:** Prevalence of behavioral NCD risk factors (%) by nationality (n = 4,500).

Variable	Nationality			
**Smoking status**	Saudis n = 3063	ANS n = 1091	South Asian n = 309	P value
Current smokers	371 (12.1)	116 (10.6)	34 (11.0)	0.46
Cigarette smoking	229 (65)	84 (76)	34 (100)	0.03
Waterpipe smoking	123 (35)	26 (24)	0 (0.0)	<0.001
**Physical activity**				
Inactive	2422 (79.1)	785 (72.0)	236 (76.4)	<0.001
**Low fruit / vegetables intake**				
Low intake < 5 servings /day	2683 (87.6)	951 (87.2)	280 (90.6)	0.71

Data are presented as number of subjects (precentage).

#### Physical inactivity

77% of the cohort reported being physically inactive Nevertheless, physical inactivity rates were higher among women and Saudi participants compared to their counterparts (p <0.001 for both comparison **Table 2**).

#### Low fruit/vegetables consumption

Only 12% of the studied population reported consuming the recommended ≥ 5 servings of fruit/vegetable per day. Nationality did not seem to have an effect on daily intake in this study’s sample. There was however a significant difference between genders, where men reported higher consumption than women (p = 0.001) (**Table 2).**

### Clinical NCD risk factors

#### Overweight/obesity

More than two thirds of the studied population was overweight or obese based on BMI. However, using the WHtR index, a higher rate of abdominal obesity (AO) was detected among the studied population as detailed in **Table 3**. Among different nationalities: South Asian population were found to have a significantly higher rate of being overweight/obese than others (p<0.001). This difference was sustained after adjusting for age (adjusted Odds Ratio; aOR = 1.93, 95% CI = 1.4–2.67).

**Table 3:** Prevalence of the clinical NCD risk factors, the clustering of NCD risk factors and 10-year cardiovascular risk scores by nationality and gender (n = 4500).

Variable	Gender			Nationalities			
	Males 1996(44%)	Females 2504 (56%)	p value	Saudis 3062(68%)	ANS 1091(24%)	South Asian 309 (7%)	p value
**Body mass index sub-categories**							
Overweight	783(39)	783(31)	<0.001	980(32)	427(39)	137(44)	<0.001
Obesity	486(24)	570(23)	0.21	679(22)	283(26)	88(29)	<0.001
Morbid obesity	202(10)	441(18)	<0.001	451(15)	154(14)	34(11)	<0.001
**Abdominal obesity**	1349(68)	1323(53)	<0.001	1960(55)	752(69)	204(66)	<0.001
**Hypertension**							
Diagnosed	484(24)	496(20)	<0.001	613(20)	256(24)	99(32)	<0.001
Undiagnosed	37(2)	18(1)	<0.001	39(1)	11(1)	4(1)	0.76
**Abnormal glucose metabolism**							
Diabetes mellitus	363(18)	445(18)	0.37	549(18)	179(16)	74(24)	0.02
prediabetes	606(30)	601(24)	<0.001	759(25)	313(29)	125(41)	<0.001
Undiagnosed DM	87(4)	98(4)	0.48	92(3)	51(5)	27(9)	0.001
Uncontrolled DM	191(53)	219(49)	<0.001	286(52)	84(47)	35(47)	<0.001
**Dyslipidaemia**							
Total cholesterol 656(37%)	592(30)	382(15)	<0.001	553(18)	306(28)	107(35)	<0.001
Triglycerides, 974 (22%)	743(37)	913(37)	0.31	1127(37)	419(38)	94(30)	0.06
LDL-C,1540 (34%)	795(38)	775(31)	<0.001	1036(34)	395(36)	95(31)	0.26
HDL-C, 1419 (32%)	990(50)	429(17)	<0.001	785(26)	454(42)	171(55)	<0.001
Undiagnosed, 271 (6%)	157(8)	114(5)	<0.001	160(5)	85(8)	22(8)	0.009
**Next 10 –year cardiovascular risk score**							
Low risk score < 10%: n = 2472(75%)	NA*	NA*	NA*	1513(75)	672(75)	218(78)	0.80
Intermediate risk score 10–20%:n = 498 (15%)	NA*	NA*	NA*	309(15)	149(17)	35(13)	0.40
High risk score >20%: n = 305 (10%)	NA*	NA*	NA*	195(10)	80(9)	27(10)	0.50

NA* not applicable; gender already included in risk score calculation. Data are showed as number of subjects (percentage).

#### High blood pressure

The overall prevalence of hypertension was 22%, with a significantly higher rate among men than women (p<0.001). The highest frequency was observed among the South Asian population, compared to Saudi and ANS populations (p<0.001). Still, after adjustment for age, South Asians showed increased risk of having hypertension of nearly two times compared to Saudi participants (aOR = 2.05, 95% CI = 1.5–2.73) (**S2 Table**).

#### Diabetes mellitus

Diagnosed diabetes mellitus was 18% of the overall sample population, with similar prevalence in both genders (**Table 3**). The prevalence of diabetes was significantly higher among South Asians (p = 0.02) compared to other nationalities, and with age adjustment they showed higher risk of being diabetic compared to Saudis (aOR = 1.46, 95% CI = 1.1–1.98). More details are shown in **(S2 Table)**.

#### Dyslipidaemia

The prevalence of different types of dyslipidaemia ranged from 22–37%. Moreover, undiagnosed dyslipidaemia was detected among 6% of overall participants as shown in **Table 3**. All types of dyslipidaemia, except high LDL-C, were found to be higher among South Asians compared with other ethnicities (p<0.001). Adjustment for age confirmed the higher risk of TC, HDL dyslipidaemia among South Asians and decreased of elevated triglycerides (**S2 Table**).

#### Clustering of NCD risk factors

The adjusted odds ratio (aOR) increased significantly with the increase of the participant’s age; for example: subjects aged 30–40 years were at an almost two-fold higher risk (aOR = 2.3, 95% CI = 1.7–3.2), and participants aged ≥50 years were at a higher risk of 11.3-fold; to have ≥3 risk factors when compared to younger age groups (19–29 years old). However, men were found to have two-fold clustering of ≥3 risk factors compared to women (aOR = 1.9, 95% CI = 1.6–2.2) **(S1 Table).**

Married participants were more likely to have clustering of ≥3 risk factors when compared to single participants (aOR = 1.5, 95% CI = 1.1–2.1). In addition, widowed and divorced participants had nearly two-fold higher risk of having ≥3 risk factors compared to singles. On the other hand, education, occupation and nationality had no conclusive evidence of association with clustering risk factors (**S1 Table)**.

#### Next 10-year cardiovascular risk score

Cardiovascular risk scores were calculated for the study population who were ≥ 30 years old (72% of the whole population), of whom 15% had intermediate and 10% had high risk scores (**Table 3**). There were no significant differences between nationalities with regards to intermediate and high risk scores (**Table 3)**.

## Discussion

In this large cohort of employees and their dependents in a Saudi university, we observed alarmingly high rates of NCD risk factors. Unhealthy dietary pattern and physical inactivity were the most prevalent factors, affecting more than 80% of the participants, followed by high rates of overweight/obesity, dyslipidaemia, diabetes and hypertension. Indeed, we think that the epidemiological profile of this worksite closely resembles that of the general population in KSA as well as some Eastern Mediterranean Region (EMR) countries [5, 7, 28–33].

It is not a surprise to find such prevalent behavioral NCD risk factors profile across the different ethnic groups included in this study, where risk factors such as unhealthy dietary pattern and physical inactivity were also reported to be high in studies performed in KSA and other EMR countries [7, 30, 34]. The only exception is tobacco use, which was reported to be quite low compared to other studies in KSA [7, 10, 35]. The effect of nutrition transition was clearly translated in the low intake of fruit/vegetables, despite a large body of evidence which indicates that high and regular fruit/vegetable consumption is usually associated with reduction in the NCD risk factors, stroke and DM occurrence [36]. Nevertheless, just above one tenth of this study's respondents consumed the recommended daily servings, which is in concordance with findings from KSA and Bahrain [7, 30, 34].

Moreover, the KSA economic transition seemed to have changed traditional lifestyle and occupational patterns to the detriment of physical activity in KSA as well as in GCC region [37]. In this study the majority of the participants (more than two thirds) reported to be physically inactive. Saudis and women were more likely to be physically inactive, which analogous to recent GCC reports [7, 28, 30].

Interestingly, the reported behavioural risk factors profile of South Asian subjects living in KSA is strongly consistent with that of Arab non-Saudi and Saudi participants in this study, but much higher than that reported from Asia [38, 39]. This similarity could be explained by acculturation theory, where migrants gradually change and adopt behaviours of host cultures [40].

We observed an alarming prevalence of obesity across the studied nationality groups. Women had a higher rate of obesity compared to men, which is comparable with other national and regional studies [33, 41]. The prevalence of obesity was more striking when measured by WHtR. By using the BMI definition, obesity rate was underestimated by nearly two-fold among this study's participants [42]. Likewise, South Asian participants were found to have higher rates of overweight/obesity than levels reported from other South-Asian and GCC studies [38, 39, 43].

One fifth of participants in our study were previously diagnosed as having hypertension, which is in agreement with previously reported estimates among university employees in KSA [7, 10], but lower than the rates previously reported from KSA and GCC countries [5, 9].

Reported prevalence of 22–30% for diabetes mellitus by recent International Diabetes Federation reports and other KSA studies (national and workplace), is comparable with our findings [7, 9, 31]. Almost one half of the studied population had abnormal glucose metabolism, where nearly 22% of them had overt diabetes mellitus, while more than one quarter of the overall studied population was found to be prediabetic, which coincides with a recent nationwide estimate of 51% [44]. After adjustment for age, South Asian individuals were estimated to have higher rates of diabetes in our work, which is much higher than the estimated prevalence reported other studies [38, 39, 43].

In this study, hyperlipidemia was reported to affect more than one third of the population, which is comparable to results reported from previous studies in KSA [7, 10, 29]. Males had higher rates of all types of dyslipidaemia in this study, which is consistent with other studies [45, 46]. South Asians residing in KSA had a higher level of lipid profile, compared to Arabs, a finding that remained high after adjusting for age. This profile is in concordance with a report from Kuwait [43], but exceeds reports from their countries of origin [38, 47]. This high prevalence could be explained by consequences of living in KSA coupled with behavioural risk factors such as PI, unhealthy diet and obesity.

Our results revealed that one quarter of the studied population had clustering of ≥3 NCD risk factors, which is much lower than that reported from a Saudi study[7]. In addition, men and those aged ≥ 50 years presented with higher levels of ≥3 clustering NCD risk factors, which is in agreement with the same Saudi report [7].

The prediction of CVD events during the following 10 years showed that one quarter of the screened population had intermediate (15%) and high-risk scores (10%). Surprisingly, no significant differences were noticed between ethnic groups in this study. These findings are higher than the most recent national study in KSA [48]. The intermediate-risk rate of this study is in agreement with a national study from United Arab Emirates (UAE), whereas the high-risk rate is double that reported from UAE [49].

## Strengths of the Study

This study is among very few in KSA which explores the prevalence of NCD risk factors among worksite staff, and thus predicts the CVR for the following 10-years. Secondly, the study represents a comprehensive survey of NCD risk factors using a standardised WHO STEPwise approach, where, data was collected by well-trained research assistants under close supervision. Finally, compared to other similar studies this is the only large scale study (n = 4500) of the working population and their families in KSA.

## Limitations of the study

An important limitation of the study is the cross-sectional design, which does not allow establishment of cause-effect relationships, especially between established risk factors and identified outcomes (whether CVD or otherwise). Secondly, the questions of some behavioural risk factors were self-reported and subject to biases as social desirability and recall bias. Thirdly, the findings of this study cannot be generalised to the entire Saudi population, as they only portray the current status of the community of King Saud University's employees and their families. Finally, notwithstanding this the Framingham coronary heart risk score calculator has been used previously in KSA and other GCC countries; however its use has not been validated on Arab ethnicities so far, thus it may have under/overestimated CVR among this study's population.

## Recommendations

Despite above limitations this is a comprehensive survey and the findings are expected to inform public health policy and practice, to take further steps towards best interventions for changing adverse behavioural patterns among King Saud University population, en route towards building an effective public health strategy to prevent CVD events, and improve cardiovascular wellness among the university staff and their dependents.

Moreover, such a rate of clustering of risk factors is high enough to justify both population and high risk based public health intervention programs and health promotion activities to prevent further increase and manage future CVD burden [50]. Behavioral intervention progamme focusing on promoting and sustaining a healthy diet and active lifestyle at the level of the university would be promising in preventing and reducing the burden of diet-related diseases [51].

## Conclusion

Results of this study reflected the high prevalence of modifiable NCD risk factors among the studied population. Such prevalent NCD risk factors can place King Saud University community at risk of considerable NCD, particularly CVD morbidity and mortality in the near future. Furthermore, this study showed that expatriates had significant negative effects on behavioral risk factors after residing in KSA; namely poor dietary practice and physical inactivity. As a result, those risk factors were quite prevalent across the ethnic groups, resembling the Saudi general population levels as delineated previously and leading to migrants' acculturation.

## Supporting Information

S1 Table Univariate analysis of the correlation of clustering NCD risk factors among studied population (4,500). (DOC)

S2 Table Age adjustment for clinical NCD risk factors among studied ethnic groups. (DOC)

S3 Table STEPS questionnaire. (DOC)
