# Supplementary material for: Non-Communicable Disease Risk Factors among Employees and Their Families of a Saudi University: An Epidemiological Study
Source: PLoS One. 2016 Nov 4;11(11):e0165036. doi: 10.1371/journal.pone.0165036 (PMC5096675; doi:10.1371/journal.pone.0165036)
Supplement: S1 Table — (DOC) [file pone.0165036.s001.doc]

**Supporting information**

**S1 Table**: Univariate analysis of the correlation of clustering NCD risk factors among studied population (4,500).

| **Variable** | **< 3 Risk Factors n(%)**  3331(74%) | **≥3 Risk Factors**  n (%)  1169 (26%) | **Adjusted OR (95% C.I)** | **p-value** |
| --- | --- | --- | --- | --- |
| **Gender** |  |  |  |  |
| Females (n=2504) | 1954(58.7) | 550(47.0) | reference |  |
| Males (n-1996) | 1377(41.3) | 619(53.0) | 1.9 (1.6- 2.2) | <0.01 |
| **Age group (years)** |  |  |  |  |
| 18-29 (n=1270) | 1186(35.6) | 84(7.2) | reference |  |
| 30-39 ( n=1168) | 972(29.2) | 196(16.8) | 2.3(1.7-3.2) | <0.01 |
| 40-49 (n= 904 ) | 602(18.1) | 302(25.8) | 5.9(4.3-8.0) | <0.01 |
| ≥50 ( n= 1158) | 571(17.1) | 587(50.2) | 11.2(8.3-15.3) | <0.01 |
| **Nationality** |  |  |  |  |
| Saudis (n=3063) | 2320(70.2) | 743(64.2) | reference |  |
| South Asians ( n=309) | 229(6.9) | 80(6.9) | 0.9 (0.7-1.2) | 0.5 |
| Arab non Saudis (n= 1091) | 757(22.9) | 334(28.9) | 1.2(1-1.4) | 0.07 |
| **Educational level** |  |  |  |  |
| Essential education (n=1203) | 929(27.9) | 274(23.4) | reference |  |
| Illiterate ( n=162) | 82(2.5) | 80(6.8) | 1.4(1-2.0) | 0.07 |
| Higher education (n=3135) | 2320(69.6) | 815(69.7) | 0.83(0.7-1.0) | 0.07 |
| **Occupation** |  |  |  |  |
| Technician (n=159) | 127(9.9) | 32(6.3) | reference |  |
| Health provider ( n=201) | 155(12.1) | 46(9.0) | 0.9(0.5-1.6) | 0.7 |
| Administrative (n=319) | 251(19.6) | 68(13.3) | 0.8(0.5-1.4) | 0.4 |
| Teaching staff (n=1113) | 748(58.4) | 365(71.4) | 0.7(0.5-1.2) | 0.2 |
| **Marital Status** |  |  |  |  |
| Single (n=863) | 806(24.2) | 57(4.9) | reference |  |
| Divorced/widowed (n=129) | 80(2.4) | 49(4.2) | 2.1 (1.3-4.0) | <0.01 |
| Married (n=3508) | 2445(73.4) | 1063(90.9) | 1.5(1.1-2.1) | 0.02 |
| **Legend**: Logistic regression model was used to adjust for the possible effect of different socio-demographic variables on clustering risk factors. Data are presented as numbers (%). | | | | |
