# Supplementary material for: Non-Communicable Disease Risk Factors among Employees and Their Families of a Saudi University: An Epidemiological Study
Source: PLoS One. 2016 Nov 4;11(11):e0165036. doi: 10.1371/journal.pone.0165036 (PMC5096675; doi:10.1371/journal.pone.0165036)
Supplement: S2 Table — (DOC) [file pone.0165036.s002.doc]

**S2 Table**: Age adjustment for clinical NCD risk factors among different ethnic groups (n=4,500)

| Variable | ANS  n=1091  aOR (95% CI) | South Asians  n=309  aOR (95% CI) |
| --- | --- | --- |
| Overweight/obesity | 1.49(1.26-1.78) | 1.93(1.40-2.67) |
| Hypertension | 1.22(1.1-1.47) | 2.05(1.54-2.73) |
| Diabetes mellitus | 0.84(0.69-1.03) | 1.46(1.11-1.98) |
| Dyslipidaemia |  |  |
| TC | 1.71(1.46-2.02) | 2.31(1.79-2.98) |
| TG | 1.02(0.88-1.17) | 0.70(0.54-0.90) |
| LDL | 1.07(0.92-1.23) | 0.82(0.64-1.06) |
| HDL | 2.05(1.77-2.37) | 3.56(2.8-4.5) |

**Legend**: Overweight/obesity ≥ 25 kg/m^2^ for Saudis and ANS and ≥23.1 for South Asian, DM if HBA1C> 6.5%, Adjusted Odds Ratio (aOR) with 95% confidence interval (CI) was calculated to each clinical NCD risk factors by considering Saudi participants as reference.
