# Supplementary material for: Non-Communicable Disease Risk Factors among Employees and Their Families of a Saudi University: An Epidemiological Study
Source: PLoS One. 2016 Nov 4;11(11):e0165036. doi: 10.1371/journal.pone.0165036 (PMC5096675; doi:10.1371/journal.pone.0165036)
Supplement: S3 Table — (DOC) [file pone.0165036.s003.doc]

**Supporting information**

S **3. STEPS questionnaire**

Interviewer name: Date of interview:

Consent has been read and obtained: Yes No

**This part is to be completed by participant**

| **Step one :Socio-economic Data** | | | | | |
| --- | --- | --- | --- | --- | --- |
| 1. **Demographic information** | | | | | |
| **Questions** | | | **Response:** Please tick one box or fill the space | | |
| 1. Gender | | | Male  Female | | |
| 1. Date of birth | | | dd/mm/yy/: / / / | | |
| 1. Age | | | ………..Year | | |
| 1. Level of education | | | PhD  Master  bachelor degree  diploma high school intermediate school  secondary school I don’t read or write  other please specify: | | |
| 1. Marital Status | | | Married Single Widowed  Divorced | | |
| 1. Nationality | | | Saudi Non-Saudi, specify:  If non-Saudi please mention your original country:  When did you move to Saudi Arabia:  ……….. | | |
| 1. King Saud University Post Title | | | Faculty  Healthcare provider  Technician  Administrative  Staff’s family member   under graduate  post graduate other specify: | | |
| 1. **Step one: Behavioral Measurements** | | | | | |
| **1-Tobacco use:** | | | | | |
| **Questions** | | | **Response:** Please add narrative/number/ mark as appropriate | | |
| **Living in Saudi Arabia (for both Saudis and non-Saudi)** | | | | | ***For Non-Saudi***  ***before coming to Saudi Arabia, please describe your smoking status*** |
| 1. Do you currently smoke any type of tobacco (cigarette, shisha, cigar, or pipe)? | | Current smoker Former smoker   Non-smoker | | | Current smoker Former smoker   Non-smoker |
| 1. If the answer “current smoker “Do you currently smoke any type of the aforementioned tobacco types? | | Yes No | | | Yes No |
| 1. How old were you when you first started smoking daily? | | Age: years | | | Age: years |
| 1. Do you remember how long ago it was? | | Week…….Month ……years …… | | | Week…….Month ..  years … |
| 1. On the average, how many of the following do you smoke each day? | | -Manufactured cigarettes:..  -Shisha……….  -Hand-rolled cigarettes:  -Pipes full of tobacco:….  -Cigars, cheroots,  cigarillos: | | | -Manufactured cigarettes:..  -Shisha……….  -Hand-rolled cigarettes:  -Pipes full of tobacco:…..  -Cigars, cheroots, cigarillos: |
| 1. During the past 12months, have you tried to stop smoking? | | Yes No | | | Yes No |
| 1. During any visit of a doctor or other health worker in the past 12 months, were you advised to quit smoking tobacco? | | Yes No | | | Yes No |
| **2- Healthy Diet** | | | | | |
| **Questions** | | | **Response:**  Please add narrative/number/ mark as appropriate | | |
| **Living in Saudi Arabia (for both Saudis and non-Saudi)** | | | | ***For Non-Saudi***  ***before coming to Saudi Arabia, please describe your Diet habits*** | |
| 1. In a typical week, how many **days** do you eat fruits? (Apple, orange, banana, dates ...etc | Number of da**ys** that you eat fruits _______ per week | | | Number of days that you eat fruits _______ per week | |
| 1. How many servings of fruit do you eat on one of those **days**? | Number of servings____  **1 serving=1 piece of any type of fruits**  **1 serving= ½ cup (40 gm)of any fruit juice**  **1 serving= 3 dates**  **1 serving= palm size of watermelon or melon** | | | Number of Servings-------  1 serving=1 piece of any type of fruits  1 serving= ½ cup (40gm)of **any fruit juice**  **1 serving= 3 dates**  **1 serving= palm size of watermelon or melon** | |
| 1. In a typical **week**, how many days do you eat vegetables? ( cooked or uncooked) | Number of **days**______ per week | | | Number of **days**______ per week | |
| 1. How many servings of vegetables do you eat a **day**? | Number of Servings_____  **1serving = 1/2cup (40gm)cooked or chopped vegetables**  **1 serving= 1cup (80gm) of raw green leafy vegetables**  **1 serving =1/2 (40gm)cup of vegetable juice** | | | Number of Servings_____  **1serving = 1/2cup (40gm)cooked or chopped vegetables**  **1 serving= 1cup (80gm)of raw green leafy vegetables**  **1 serving =1/2 cup (40gm)of vegetable juice** | |
| 1. What type of oil / fat is most often used in a meal preparation at your household? | Vegetable oil butter or ghee Margarine others | | | Vegetable oil butter or ghee Margarine others | |
| 1. On the average, how many meals per week do you eat that were not prepared at home? By meal, I mean breakfast, lunch or dinner ( such as pizza, hamburgers, French fries and deep-fried chicken) | Number of meals______ per week  I don’t know -------------- | | | Number of meals______per week  I don’t know -------------- | |

| **3- Physical activity** | | | | | | | | | |
| --- | --- | --- | --- | --- | --- | --- | --- | --- | --- |
| **Questions** | **Response:**  Please add narrative/number/ mark as appropriate | | | | | | | | |
| **Living in Saudi Arabia (for both Saudis and non-Saudi)** | | | **For Non-Saudi**  **before coming to Saudi Arabia, please describe your physical activities** | | | | | | |
| **Work** | | | | | | | | | |
| 1. Does your work involve vigorous-intensity activity that causes large increases in breathing or heart rate like (carrying or lifting heavy loads, digging or construction work)for at least 10 minutes continuously? | Yes No | | | | | | | | Yes No |
| 1. In typical week, on how many days do you do vigorous-intensity activities as part of your work? | Number of days: | | | | | | | | number of days: |
| 1. How much time do you spend doing vigorous-intensity activities at work on a typical day? | Hours:…………  Minutes:……… | | | | | | | | Hours:…………  Minutes:……… |
| 1. Does your work involve moderate-intensity activity that causes small increases in breathing or heart rate such as brisk walking (or carrying light loads) for at least 10 minutes continuously? | Yes No | | | | | | | | Yes No |
| 1. In typical week, on how many days do you do moderate -intensity activities as part of your work? | Number of days: | | | | | | | | Number of days: |
| 1. How much time do you spend doing moderate -intensity activities at work on a typical day? | Hours:…………  Minutes:…… | | | | | | | | Hours:………  Minutes:…  None |
| **Travel to / from places** | | | | | | | | | |
| 1. Do you walk or use bicycle (pedal cycle) for at least 10 minutes continuously to get to / from work /places? | Yes No | | | | | | | | Yes No |
| 1. In a typical week, on how many days do you do walk or bicycle for at least 10 minutes continuously to get to/ from work/ places? | Number of days:   None | | | | | | | | Number of days:   None |
| 1. How much time do you spend walking or bicycling for moving on a typical day? | Hours:………………  Minutes:…………… | | | | | | | | Hours:……………  Minutes:………… |
| **Recreational activities** | | | | | | | | | |
| 1. Do you do any vigorous-intensity sports, fitness or recreational (leisure) activities that cause large increases in breathing or heart rate like (running or football) for at least 10 minutes continuously? | Yes No | | | | Yes No | | | | |
| 1. In a typical week, on how many days do you do vigorous-intensity sports, fitness or recreational (leisure) activities? | Number of days: | | | | number of days: | | | | |
| 1. How much time do you spend doing vigorous-intensity sports, fitness or recreational activities on a typical day? | Hours:………………  Minutes:…………… | | | | Hours:………………  Minutes:…………… | | | | |
| 1. Do you do any moderate-intensity sport, or fitness or recreational (leisure) activities that cause small increases in breathing or heart rate such as brisk walking (cycling, swimming, volleyball) for at least 10 minutes continuously? | Yes No | | | | Yes No | | | | |
| 1. In typical week, on how many days do you do moderate -intensity sports, fitness or recreational activities? | Number of days: | | | | Number of days: | | | | |
| 1. How much time do you spend doing moderate -intensity sports, fitness or recreational activities on a typical day? | Hours:………………  Minutes:…………… | | | | Hours:………………  Minutes:…………… | | | | |
| 1. **Medical History** | | | | | | | | | |
| 1. **History of Raised Blood Pressure** | | | | | | | | | |
| 1. Have you ever had your blood pressure measured by a doctor or nurse | | | | | | | | Yes No | |
| 1. Have you ever been told by a doctor or other health worker that you have high blood pressure (hypertension)? | | | | | | | | Yes No | |
| 1. Have you been told so in the past 12 months? | | | | | | | | Yes No | |
| Are you currently receiving any of the following management /advice for high blood pressure prescribed by doctor? | | | | | | | | | |
|  | 1-Medication that you have taken in the past 2 weeks | | | | | Yes No | | | |
|  | 2- Advice to reduce salt intake | | | | | Yes No | | | |
|  | 3- Advice or treatment to lose weight | | | | | Yes No | | | |
|  | 4- Advice or treatment to stop smoking | | | | | Yes No | | | |
|  | 5- Advice to start or do more exercise | | | | | Yes No | | | |
| 1. Have you ever seen a traditional healer for raised blood pressure? | | | | | | Yes No | | | |
| 1. Are you currently taking any herbal or traditional remedy for your high blood pressure | | | | | | Yes No | | | |
| 1. Is there a family history of hypertension or do one of your parents, brothers or sisters suffer from high blood pressure (hypertension) | | | | | | Yes No | | | |
| **History of Diabetes** | | | | | | | | | |
| 1. Have you ever had your blood sugar measured by a doctor or health worker? | | | | | | | Yes No | | |
| 1. Have you ever been told by a doctor or other health worker that you have raised blood sugar or diabetes? | | | | | | | Yes No | | |
| 1. Have you been told so in the past 12 months? | | | | | | | Yes No | | |
| **Are you currently receiving any of the following treatment /advice for high blood glucose prescribed by a doctor?** | | | | | | | | | |
|  | 1-Insulin | | | | | | | | Yes No |
|  | 2- Medication that you have taken in the past 2 weeks | | | | | | | | Yes No |
|  | 3-Special prescribed diet | | | | | | | | Yes No |
|  | 3- Advice or treatment to lose weight | | | | | | | | Yes No |
|  | 4- Advice or treatment to stop smoking | | | | | | | | Yes No |
|  | 5- Advice to start or do more exercise | | | | | | | | Yes No |
| 1. Have you ever seen a traditional healer for diabetes or raised blood glucose? | | | | | | | | | Yes No |
| 1. Are you currently taking any herbal or traditional remedy for your diabetes? | | | | | | | | | Yes No |
| 1. Have you ever been diagnosed as having Left Ventricular Hypertrophy | | | | | | | | | Yes No |
| 1. Have you ever been diagnosed as having Chronic Kidney Disease | | | | | | | | | Yes No |
| 1. Have you ever been diagnosed as having Atrial Fibrillation | | | | | | | | | Yes No |
| 1. Have you ever been diagnosed as having Rheumatoid Arthritis | | | | | | | | | Yes No |
| **3-Family History** | | | | | | | | | |
| 1. Has there been any death in your 1st degree family members before 60 years ( father , mother , brother, sister)due to heart attack myocardial infarction( MI) or stoke | | | | Ye No  If yes, please indicate the relationship…… | | | | | |
| 1. Has there been any death in your 2nd degree family members before 60 years (grandfather , grandmother, uncle , aunt)due to heart attack myocardial infarction ( MI) or stoke | | | | Yes No  If yes, please indicate the relationship…….. | | | | | |
| **Step two: Physical measurements** | | | | | | | | | |
| **Name of healthcare provider who took the physical measurements:** | | | | | | | | | |
| 1. Height | | (cm) | | | | | | | |
| 1. Weight | | (kg) | | | | | | | |
| 1. Hip circumference | | (cm) | | | | | | | |
| 1. Waist circumference | | (cm) | | | | | | | |
| 1. Neck Circumference | | (cm) | | | | | | | |
| 1. Blood pressure measured twice in : sitting position and left arm with 5 minutes interval between the two readings | | First reading :   - Systolic Blood Pressure : ____ ( mmHg) - Diastolic Blood Pressure:_____ ( mmHg) | | | | | | | |
|  |  | Second reading :   - Systolic Blood Pressure : ____ ( mmHg) - Diastolic Blood Pressure:_____ ( mmHg | | | | | | | |
| 1. During past 2 weeks , have you been treated for high blood pressure with medications prescribed by doctor | | Yes No | | | | | | | |
| **Step three: Biochemical measurements** | | | | | | | | | |
|  **Previously ordered**  Date: |  **Ordered now Date:** All HBA1c | | | | | | | | |
| 1. Was the patient fasting for the last 12hours | | | Yes No | | | | | | |
| 1. Today, have you taken insulin or other medication that have been prescribed by a doctor for diabetes? | | | Yes No | | | | | | |
| 1. Fasting blood glucose | | | mmol/l | | | | | | |
| 1. During the past 2 weeks have you been treated for raised cholesterol with medication prescribed by a doctor? | | | Yes No | | | | | | |
| 1. Triglycerides (TG) | | | mmol/l | | | | | | |
| 1. Total Cholesterol | | | mmol/l | | | | | | |
| 1. High-density lipoprotein (HDL) | | | mmol/l | | | | | | |
| 1. Low-density lipoprotein (LDL) | | | mmol/l | | | | | | |
| 1. Glycosylated haemoglobin(HBA1c) | | | % | | | | | | |

3
